# Exploring the Feasibility of the Creyos Cognitive Assessment Tool Among Patients With Heart Failure

**DOI:** 10.7759/cureus.97158

**Published:** 2025-11-18

**Authors:** Joann Varickanickal, Steven Chu, Richard H Swartz, Todd Murray, Rima Styra, Sydni G Paleczny, Adrian M Owen, Peter C Austin, Anne Simard, Louise Y Sun, Filio Billia, Karem Abdul-Samad, Heather J Ross, Douglas S Lee

**Affiliations:** 1 Cardiology, University Health Network, Toronto, CAN; 2 Neurology, Sunnybrook Health Sciences Centre, Toronto, CAN; 3 Psychiatry, University Health Network, Toronto, CAN; 4 Neuroscience, Western University, London, CAN; 5 Statistics, University of Toronto, Toronto, CAN; 6 Anesthesiology, Stanford University School of Medicine, Palo Alto, USA

**Keywords:** cognitive assessment, cognitive function, digital tool, feasibility, heart failure

## Abstract

Objective

The objective of this study is to explore the feasibility of the Creyos Cognitive Assessment Tool (Creyos, Toronto, Canada) as a cognitive testing instrument for patients with heart failure (HF).

Background

Identifying appropriate methods for cognitive testing among patients with HF is important to enable further study of their high prevalence of cognitive decline.

Methods

A total of 40 participants, composed of 30 outpatients with HF (75%) and 10 patients without HF (25%), were asked to complete all 12 online Creyos tests, which collectively measure short-term memory (STM), reasoning, concentration, and verbal ability. The participants had the option to complete the study at home or in person at the hospital if they did not have computer/tablet access. We explored completion rate and completion time and evaluated how our participants compared to a sex/age-matched healthy population (obtained from Creyos).

Results

Among 45 patients who consented to participate, five withdrew, and 40 (89%; median age: 68 {interquartile range (IQR): 59-76} years; 78% men) completed all 12 tests of cognition. Overall, the participants had a positive experience with the tool, with a median time for completion of 25.9 minutes. When compared to age- and sex-matched norms, 73% of participants with HF showed marked impairment (>1.5 standard deviations {SDs}), and 93% showed mild impairment (>1 standard deviation) on at least one test. Individuals with HF performed worse on the reasoning and verbal ability domains but better on the STM domain when compared to the age/sex-matched healthy population data provided by Creyos.

Conclusion

It is feasible to use Creyos as a cognitive assessment tool in the population with HF, and it may enable further exploration of the connections between cognitive function and heart failure.

## Introduction

Over the lifetime of patients with heart failure (HF), there is a high prevalence of cognitive impairment (CI), estimated at 43% [1]. Cognitive decline in patients with HF is multifactorial but may be attributed, in part, to reduced blood flow [2,3]. Potential mechanisms include systemic inflammation, leading to the breakdown of the blood-brain barrier, causing a decline in cognitive function [4-8]. There are many potential confounders in the assessment of cognitive function among patients with HF, such as comorbidities and medications [9,10]. Patients with other cardiac conditions, such as atrial fibrillation and aortic valve stenosis, also experience cognitive decline [11,12].

The social determinants of health (SDOH) also contribute to cognitive impairment and impact how patients with HF manage their symptoms [9,10]. For example, education and health literacy levels may impact performance on cognitive assessments [9]. Social support also plays a role in providing care for patients with HF with CI [9,10]. More research is needed to explore the relationship between the social determinants of health and HF-related cognitive decline. Existing literature exploring the impact of SDOH on HF outcomes does not explore cognitive decline as an outcome [13,14].

After an HF hospitalization, 30-day readmissions occur twice as often in patients with cognitive impairment compared to those without the condition [2]. The greater risk of hospitalization in those with HF and cognitive impairment may be the result of suboptimal self-care, because of (i) difficulties adhering to recommendations, forgetfulness, and decreased ability to learn; (ii) decline in language function; and (iii) inability to recognize and process worsening HF symptoms due to reduced executive function and psychomotor speed [3-5].

There is a need to implement initiatives for the prevention and earlier detection of cognitive impairment in the adult population with HF, as highlighted by the Heart and Stroke Foundation of Canada and the 2024 Canadian Stroke Best Practice Recommendations (CSBPR) [10,15]. A scientific statement made by the Heart Failure Society of America (HFSA) in 2024 also noted the need for a better understanding of the pathophysiology of cognitive impairment among patients with HF and the further exploration of the link between heart-brain health and the social determinants of health [8]. In addition, the scientific statement emphasized the importance of detecting and characterizing cognitive impairment in individuals with HF at an earlier stage [8]. Both the 2022 American Heart Association (AHA)/American College of Cardiology (ACC)/HFSA guidelines for the management of heart failure and the CSBPR provide suggestions for screening tools, most of which are traditionally paper-and-pen tests [16,10].

To address some of these gaps, it is necessary to identify an appropriate cognitive assessment tool that can be used in patients with HF. The objective of this study was to explore the feasibility of the Creyos Cognitive Assessment Tool (Creyos, Toronto, Canada) as a cognitive testing instrument for the population with HF. To measure feasibility, we focused on participant completion rate, completion time, and experience. We also explored how our data compared to normative data.

## Materials and methods

Cognitive tests

In this study, we used the Creyos Cognitive Assessment Tool, an online tool that uses 12 tests to measure aspects of short-term memory (STM), concentration, reasoning, and verbal ability [17]. We selected Creyos for this study as it could be completed remotely and without a trained administrator. The tests were completed on a computer or tablet. Each test started with a tutorial, where the participant practiced the tasks before starting the test. Completing all 12 tests takes about 24-36 minutes on average. The test was also available in other languages, including French and Spanish; however, language availability was not relevant in this population. In addition, the Creyos battery has been used in several large-scale studies to assess the effects of sleep [18], COVID-19 [19], exercise and video gaming [20], and functional neuroimaging in healthy adults [17]. The tests have been validated for use in the general population across the lifespan [21] and in studies with children [22,23] and older adults [24,25]. The tests have also been validated in several clinical cohorts, such as patients with traumatic brain injury (TBI) [26], neurodegenerative populations [27], ICU patients [28], and cardiac patients [29]. Until now, it has not been used to test cognition among adults living with HF.

Participant recruitment and data collection

We recruited a convenience sample of 40 patients, 30 patients with HF (75%) and a control group with 10 patients without HF (25%), from the outpatient heart function clinic at Toronto General Hospital in Ontario, Canada, from February to August 2024, using the inclusion/exclusion criteria in Table 1. We included patients with HF with reduced ejection fraction (HFrEF), HF with preserved ejection fraction (HFpEF), or HF with midrange ejection fraction (HFmEF). Controls were composed of general cardiology patients (i.e., patients without HF). Since this was a feasibility study, the sample size was chosen pragmatically based on limited recruitment resources and the pilot study rule of thumb, which is about 12 participants per arm [30]. This was not intended to yield precise estimates of completion rate or statistical power.

**Table 1:** Participant inclusion and exclusion criteria. HF, heart failure; HFrEF, heart failure with reduced ejection fraction; HFmEF, heart failure with midrange ejection fraction; HFpEF, heart failure with preserved ejection fraction; NYHA, New York Heart Association classification

Inclusion Criteria: Patients With HF	Inclusion Criteria: Patients Without HF (Control Group)	Exclusion Criteria
Patients with heart failure over the age of 18 years old	Individuals aged 18 years and older without an active HF diagnosis	Non-English-speaking individuals
Any HF type (HFrEF, HFpEF, or HFmEF)		
Any NYHA	Able to independently complete an online cognitive assessment using a computer or a tablet (with or without a caregiver or research staff to help provide access to the test)	Diagnosed with dementia and requiring full-time support for basic physical activities
Able to independently complete an online cognitive assessment using a computer or a tablet (with or without a caregiver or research staff to help provide access to the test)		

Informed written consent was obtained from all participants. The study was approved by the Research Ethics Board of University Health Network (approval number: 23-5101). The participants had the option of asking a family member, friend, or member of the research team for help with accessing the tests or clarifying the instructions.

Most participants were emailed a unique URL and completed the tests at home. For those who did not have an email address or computer/tablet at home, they completed the test in person with a member of the research team at Toronto General Hospital. The participants who completed the Creyos tests at home were asked to complete them within two weeks. Once the participants started the tests, they had 24 hours to complete them. This was done to allow some flexibility for the participants who could not complete all 12 tests in one sitting. When the participants did not complete the tests within 1-2 weeks of receiving the link, a member of the research team sent a reminder via email and/or phone. The participants were given the option to withdraw from the study at any time by contacting the research team to inform them of their decision.

Each participant completed a demographic form before starting the Creyos tests, providing information about their age, gender, English fluency, and ethnicity (Appendices). After completion, each participant completed a Likert evaluation survey, based on the system usability scale, to provide feedback about the Creyos tool (Appendices) [13]. Surveys were sent to the participants immediately after completing the Creyos tests.

In addition, we accessed the participant’s electronic medical record for history of stroke/transient ischemic attack (TIA), hypertension, diabetes, high cholesterol, history of smoking, atrial fibrillation/flutter, HF type, and ejection fraction (EF) at the time of appointment (or results closest to appointment date). There was no missing data in this study.

Data analysis

Statistical analyses were performed using Microsoft Excel (Microsoft Corp., Redmond, WA) or R statistical software (R Foundation for Statistical Computing, Vienna, Austria). Baseline demographic and clinical characteristics were summarized using means and standard deviations (SD) or medians and interquartile range (IQR) for continuous variables, as appropriate. Proportions were used to summarize categorical data.

A one-sample t-test was conducted to evaluate the differences between patients with heart failure and a healthy population sample from Creyos. Our scores were compared to the normative scores provided by Creyos, which is made up of a group of over 85,000 participants (referred to as Creyos normative scores) [14]. Individuals from this database were age- and sex-matched to our participants with HF data age bins. The normative data provided by Creyos was collected as part of a large-scale public study examining the properties of the Creyos cognitive tasks, with the aim of better understanding human intellectual ability [17]. Since then, multiple newer published studies have contributed to the database [18,20]. Over 85,000 volunteers have contributed to the Creyos Health database, and the databases provide norms for ages six and up. The Creyos Health normative database is designed to be representative of the general population; more information on how the norms were collected is detailed in an online guide [31]. Additional data collection is ongoing, and the normative database may occasionally be updated to narrow age blocks, increase the number of people in each block, incorporate additional variables into reports, and/or better represent the current population. However, despite the major impact of cognitive impairment in patients with heart failure, the feasibility of using the Creyos battery in this population has not been previously described.

Patient performance across the reasoning, verbal ability, and short-term memory cognitive domains was assessed using loading factors, which involve multiplying the Z-score of each test by a coefficient reflecting the domain’s significance [9,15]. Loading factors were derived from Creyos, as outlined in a previous study [17]. The concentration domain was considered a theoretical domain; therefore, as a Z-score could not be calculated, it was not included in further analysis. Consequently, a patient’s overall score in each cognitive domain was derived from the total scores of all 12 tests [16]. We classified impairment from standardized Creyos Z-scores (mean = 0; SD = 1). Our primary definition used the composite score (average of all test Z-scores), with mild impairment defined as >1 SD below the norm mean and marked as >1.5 below the norm mean. We also applied a single-test threshold and reported both participant-level proportions under these definitions in our preliminary analysis. Effect sizes for group comparisons were quantified using Cohen’s d, with 95% confidence intervals [32]. Statistical significance was set at a two-sided alpha = 0.05.

Patient evaluation surveys, administered following the Creyos cognitive testing, were analyzed to assess the overall testing experience. Responses were summarized using counts and appropriate statistical measures. Additionally, the internal consistency of the survey items was evaluated using Cronbach’s alpha [33].

Due to the exploratory nature of this feasibility study, we did not apply adjustments (i.e., Bonferroni/Holm) for multiple comparisons, and results should be interpreted with caution and more so as hypothesis-generating.

Patient partner participation

The study was co-designed with a person with lived experience (PWLE), who tested the Creyos tool and provided suggestions to improve accessibility for the participants. The PWLE attended and contributed to our team meetings, ensuring that a patient lens was brought to ongoing discussions. They also reviewed the study results and this manuscript.

## Results

Recruitment and participation characteristics

**Figure 1:**
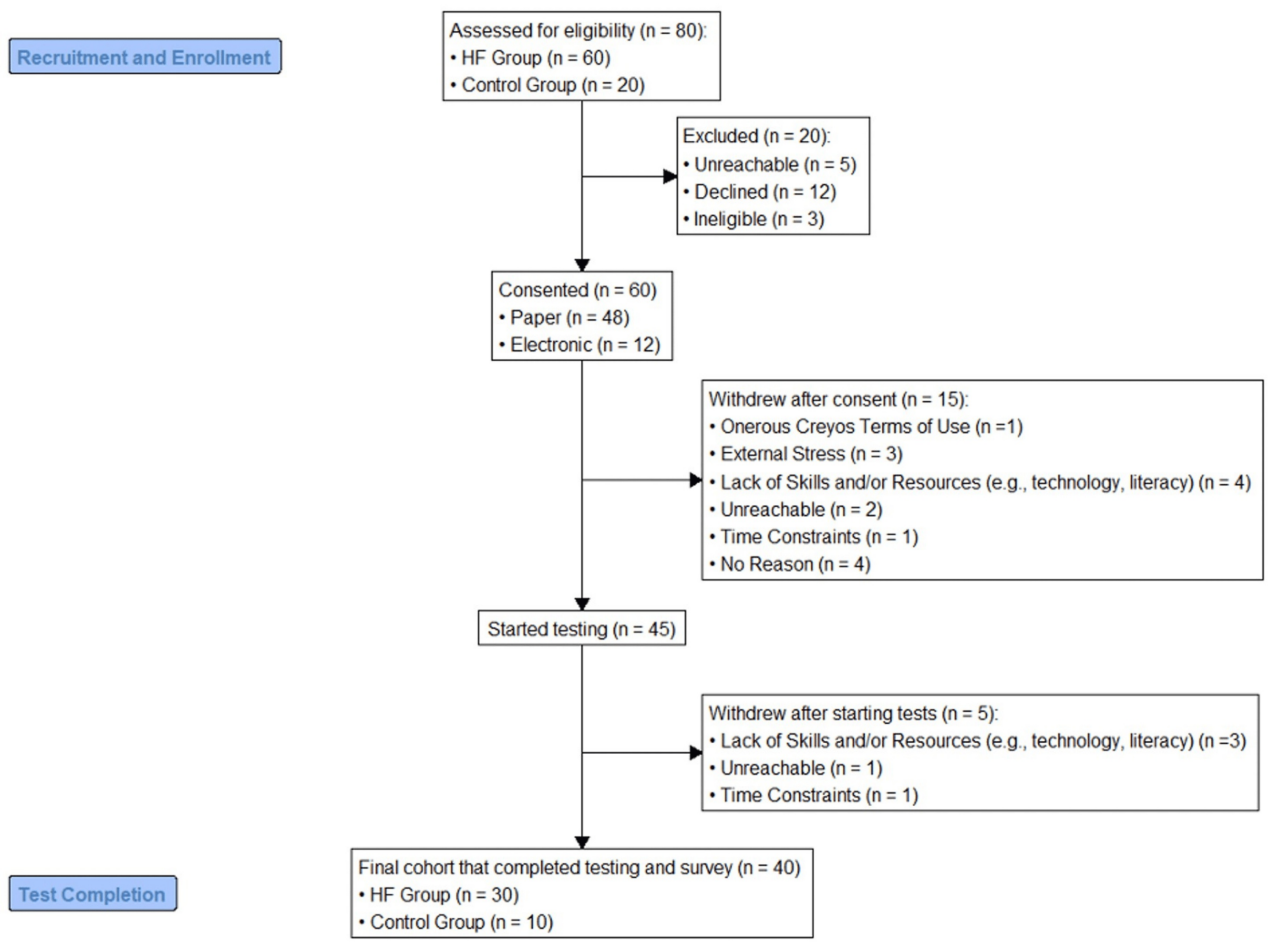
Flow chart of participant recruitment, enrollment, and study completion. HF: heart failure

Of the 80 eligible patients approached at University Health Network, 60 (75%) consented to participate. Of those consented, 45 (71%) completed at least part of the Creyos battery test at baseline, five (11%) eventually withdrew from the study, and 40 participants completed all 12 tests, along with the evaluation survey, resulting in a completion rate of 89%. The target cohort of 40 participants, 30 with HF (HF group) and 10 without HF (control group), was successfully selected from the remaining 45 participants (five of whom withdrew). The five that were partial completions were withdrawn and not included in the analysis. The 40 participants were the final analysis set. Controls were non-HF cardiology controls because this allowed for a comparison between patients with American Heart Association stage C HF (symptomatic) and those with stage B HF (heart disease without HF symptoms). Comparison with healthy controls would be too different to be a meaningful control. Details of recruitment and enrollment are illustrated in the flowchart (Figure 1). The median time (IQR) for completion (excluding the time it took to complete the test tutorials) was 25.9 (3.5) minutes. There was no significant difference in completion time among both groups. Of the 40 participants, 31 were men (77.5%), and the median age was 68 years (IQR: 59-76). Within the HF group, 19 patients (63%) had HFrEF, and 11 patients (37%) had HFmEF or HFpEF. Participant characteristics for both the HF and control groups are displayed in Table 2.

**Table 2:** Baseline characteristics for control and HF groups. *Median (IQR); n (%). **Wilcoxon rank sum test; Fisher’s exact test. Fib, fibrillation; PAF, persistent atrial fibrillation; HF, heart failure; HFrEF, heart failure with reduced ejection fraction; HFmEF, heart failure with midrange ejection fraction; HFpEF, heart failure with preserved ejection fraction

Characteristic	Total, N = 40*	Control, N = 10*​	HF, N = 30*​	P-value**​
Age ​(years)	68 (59, 76)	63 (50, 64)​	71 (59, 78)​	0.032
Sex				>0.900
Female	9 (23%)	2 (20%)	7 (23%)	
Male	31 (78%)	8 (80%)	23 (77%)	
Racial/ethnic group​				0.700
White	32 (80%)	10 (100%)	22 (73%)	
Black	3 (7.5%)	0 (0%)	3 (10%)	
Asian	2 (5%)	0 (0%)	2 (7%)	
Other	3 (7.5%)	0 (0%)	3 (10%)	
Hypertension	17 (43%)	4 (40%)	13 (43%)	>0.900
Diabetes (any type)	5 (13%)	0 (0%)	5 (17%)	0.300
High cholesterol	9 (23%)	4 (40%)	15 (50%)	>0.900
Smoking history				0.500
Current/former	17 (43%)	3 (30%)	14 (47%)	
Never	23 (57%)	7 (70%)	16 (53%)	
Atrial Fib/flutter				0.043
Chronic/PAF	13 (33%)	3 (30%)	10 (33%)	
No	27 (67%)	7 (70%)	20 (67%)	
HF type				<0.001
HFrEF	19 (47.5%)	0 (0%)	19 (63%)	
HFmEF	5 (12.5%)	0 (0%)	5 (17%)	
HFpEF	6 (15%)	0 (0%)	6 (20%)	

Participant feedback

All 40 participants completed the 12 questions on the evaluation survey to provide feedback on the usability of the tool. The feedback from the evaluation survey was positive overall. Some of the relevant items are listed in Table 3 (full evaluation survey is in the Appendices). Thirty-four (85%) of the participants found the tool easy to access and start, seven (18%) requested assistance from a more technical person to be able to do the test, and 33 (83%) felt that they were able to complete all the tests within a reasonable amount of time. However, five (11%) participants withdrew from the study after starting the tests, indicating that the time and technological skills required to complete the study were a barrier (Figure 1).

**Table 3:** Participants’ answers to evaluation survey. SD: standard deviation

	Mean (SD)	95% Confidence Interval
It was easy to access the test and get started.	4.3 (0.9)	4.0, 4.6
I thought this tool was easy to use.	3.9 (0.9)	3.7, 4.25
I needed the support of a more technical person to be able to do this test.	1.9 (1.2)	1.6, 2.4
I think most people would learn to use this tool very quickly.	3.8 (0.7)	3.6, 4.0
I felt very confident using the tool.	3.9 (0.9)	3.6, 4.2
I was able to complete all the tests within a reasonable amount of time.	3.9 (0.9)	3.7, 4.2

In addition to the standardized survey questions, the participants had the opportunity to provide general comments and feedback on how the research team could improve the test experience and online Creyos testing overall. Most participants responded positively or had no additional feedback, while some noted flaws and limitations with the test itself and the execution of the study. For example, a few participants noted that some of the test instructions were confusing and could use clarification. In addition, some elements of the tool were difficult to see, making it difficult for participants with poor eyesight. The participants also noted difficulties they experienced due to a lack of technological skills, with some noting that they would not have been able to complete the test without help.

Cognitive assessment

**Figure 2:**
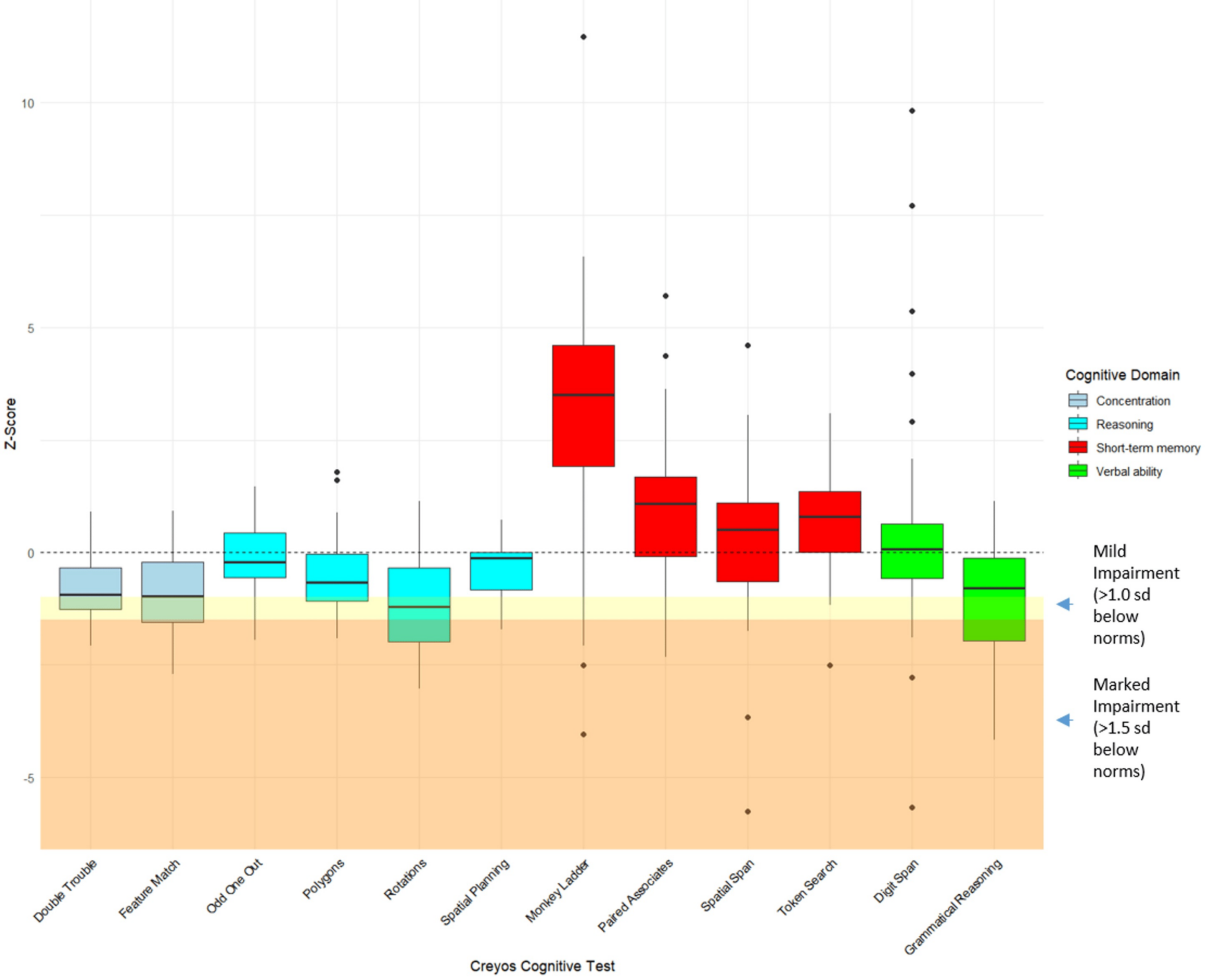
Participants with HF Z-score Creyos test performance (adjusted for age and sex). Patients with HF Creyos test performance adjusted for age and sex, indicated by Z-score, compared to healthy Creyos norms (mean = 0) on each of the 12 Creyos cognitive tests at baseline. All tests except Digit Span, Polygons, and Odd One Out showed a significant difference in mean Z-score compared to zero (p-value of <0.05); N = 30. HF, heart failure; SD, standard deviation

All participants with HF (n = 30) completed each of the 12 Creyos tests. On average, these participants performed significantly worse than age- and sex-matched normative values on tests assessing concentration (Double Trouble and Feature Match), verbal reasoning (Grammatical Reasoning), visuospatial processing (Polygons), mental rotations (Rotations), and planning ability (Spatial Planning) (Table 4). Of the 30 participants with HF, 22 (73%) demonstrated marked impairment (>1.5 SDs below age- and sex-matched to the Creyos normative scores) on at least one test, and 28 (93%) showed mild impairment (>1.0 SD) on at least one test (Figure 2).

**Table 4:** Baseline cognitive performance of participants with HF relative to healthy norms. HF, heart failure; CI, confidence interval

Cognitive Test	Mean Z-score	P-value	Cohen’s d (95% CI)
Digit Span	0.59	0.290	0.20 (-0.17, 0.56)
Double Trouble	-0.82	<0.001	-1.19 (-1.65, -0.71)
Feature Match	-0.87	<0.001	-0.88 (-1.29, -0.45)
Grammatical Reasoning	-1.03	<0.001	-0.77 (-1.17, -0.35)
Monkey Ladder	3.07	<0.001	1.02 (0.57, 1.46)
Odd One Out	-0.11	0.495	-0.13 (-0.48, 0.23)
Paired Associates	1.24	<0.001	0.74 (0.33, 1.14)
Polygons	-0.42	0.036	-0.40 (-0.77, -0.03)
Rotations	-1.07	<0.001	-0.84 (-1.25, -0.42)
Spatial Planning	-0.34	0.007	-0.54 (-0.91, -0.15)
Spatial Span	0.42	0.277	0.20 (-0.16, 0.56)
Token Search	0.61	0.013	0.48 (0.10, 0.86)

When analyzing the cognitive testing results across different HF types (HFpEF/HFmEF versus HFrEF), a significant difference was observed only in the Digit Span test (part of the verbal ability domain), with HFrEF patients performing better on average (Table 5). There were no significant differences in cognitive domains between the HF types (Table 6).

**Table 5:** Cognitive performance of HFpEF/HFmEF versus HFrEF (mean scores). HFrEF, heart failure with reduced ejection fraction; HFmEF, heart failure with midrange ejection fraction; HFpEF, heart failure with preserved ejection fraction; CI, confidence interval

	HFpEF/HFmEF (n = 11)	HFrEF (n = 19)	P-value	Cohen’s d (95% CI)
Digit Span	4.55	6.79	0.012	-0.92 (-1.69, -0.13)
Double Trouble	5.73	10.21	0.211	-0.43 (-1.17, 0.33)
Feature Match	76.09	80.00	0.736	-0.13 (-0.87, 0.61)
Grammatical Reasoning	9.09	10.74	0.506	-0.26 (-1.00, 0.49)
Monkey Ladder	6.09	6.68	0.392	-0.29 (-1.04, 0.46)
Odd One Out	9.00	9.11	0.933	-0.03 (-0.77, 0.71)
Paired Associates	3.64	4.05	0.217	-0.42 (-1.17, 0.33)
Polygons	35.09	25.21	0.258	0.46 (-0.30, 1.21)
Rotations	34.27	41.84	0.599	-0.21 (-0.96, 0.53)
Spatial Planning	10.18	13.47	0.199	-0.56 (-1.31, 0.20)
Spatial Span	4.82	4.53	0.543	0.21 (-0.54, 0.95)
Token Search	5.36	6.37	0.163	-0.57 (-1.32, 0.19)

**Table 6:** Cognitive domain performance of HFpEF/HFmEF versus HFrEF (mean domain Z-scores). HFrEF, heart failure with reduced ejection fraction; HFmEF, heart failure with midrange ejection fraction; HFpEF, heart failure with preserved ejection fraction; STM, short-term memory

	HFpEF/HFmEF	HFrEF	P-value
Reasoning	-1.86	-2.19	0.449
STM	-1.59	-1.10	0.267
Verbal ability	-1.24	-0.39	0.052

**Figure 3:**
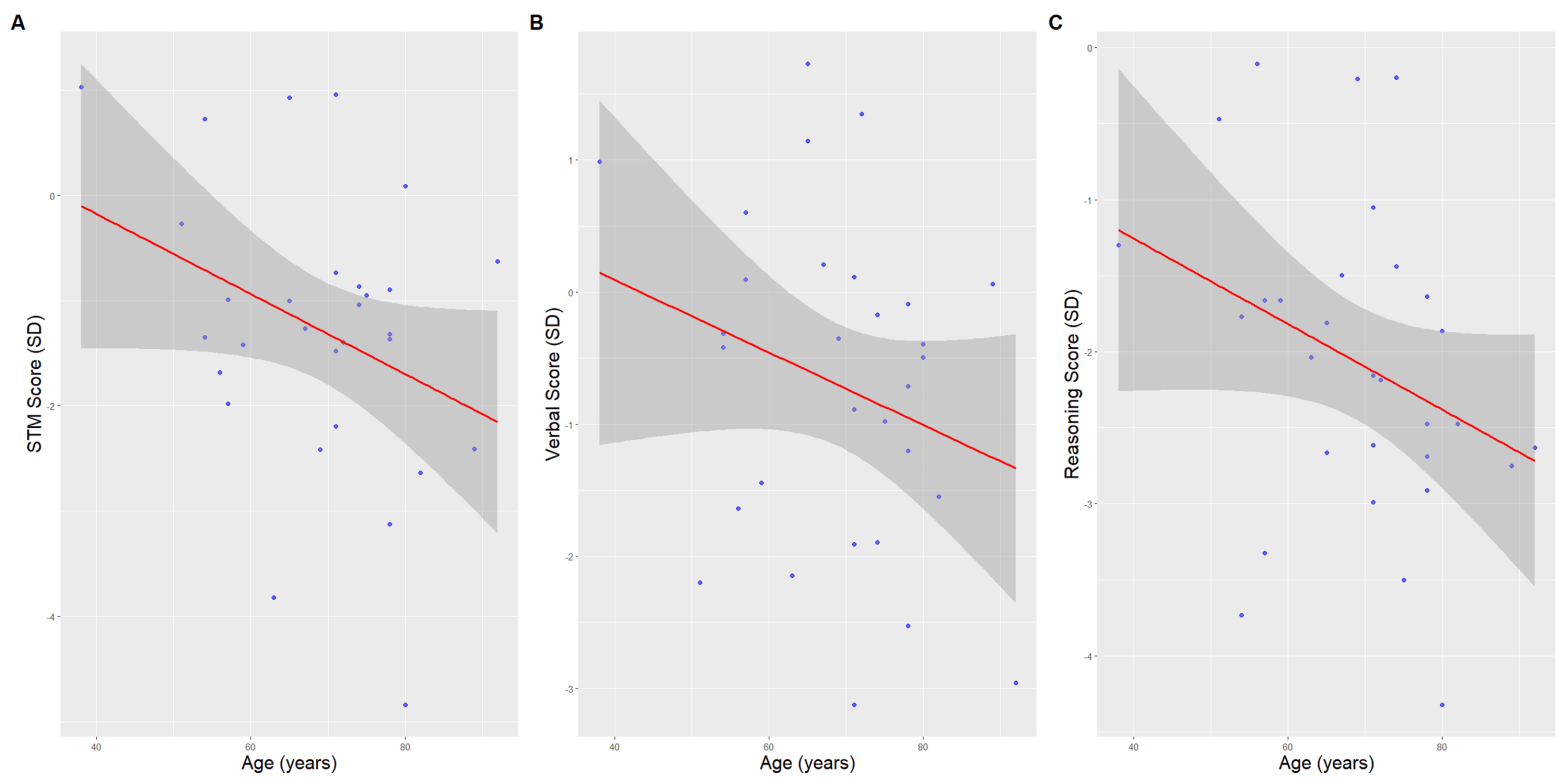
Cognitive scores (SD units) by age for patients with HF. (A) STM score by age. (B) Verbal score by age. (C) Reasoning score by age. Lines show predicted mean scores with 95% confidence intervals. STM, short-term memory; SD, standard deviation; HF, heart failure

**Figure 4:**
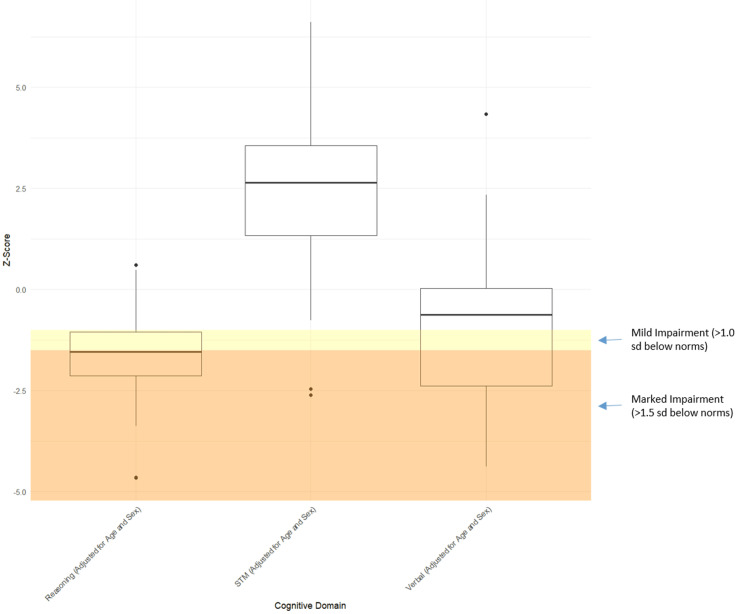
Cognitive domain performance for participants with HF. Individual patient Creyos cognitive domain performance, indicated by Z-score adjusted for age and sex and compared to healthy Creyos norms on three cognitive domains. All domain scores showed a significant difference in mean Z-scores compared to zero (p-value of <0.05); N = 30. HF, heart failure; STM, short-term memory; SD, standard deviation

In the analysis of cognitive domain scores, there was a decline in domain scores with age for short-term memory (slope = -0.039; p = 0.058), verbal ability (slope = -0.027; p = 0.178), and reasoning (slope = -0.028; p = 0.090), as shown in Figure 3. Therefore, subsequent analyses were adjusted for age in addition to sex (Table 7). Twenty-three out of the 30 (77%) patients with HF exhibited marked impairment in the reasoning domain (Z-scores of >1.0 SD below the norm), while 14 out of the 30 (47%) showed similar impairment in the verbal ability domain (Figure 4). Conversely, 26 out of the 30 (87%) patients with HF performed better than age- and sex-matched Creyos normative scores in the short-term memory domain, aligning with the observed test results (Figure 4).

**Table 7:** Ordinary least squares regression of domain scores on age and sex. STM, short-term memory; Coef, regression coefficient; SE, standard error

Regression parameter​	Score​	Coef​	SE​	P-val​ue
Age​	STM​	-0.039​	0.020​	0.058​
	Verbal​	-0.027​	0.020​	0.178​
	Reasoning​	-0.028​	0.016​	0.090​
Sex (male)​	STM​	0.647​	0.550	0.250​
	Verbal​	-0.204​	0.543​	0.710​
	Reasoning​	-0.038​	0.443​	0.933​

## Discussion

The objective of this study was to explore the feasibility of Creyos as a cognitive testing instrument for the patient population with HF. Dementia is associated with higher mortality and hospitalizations, and the early diagnosis of cognitive impairment is critically important. Standard methods of assessing cognition, such as Mini-Cog, Mini-Mental State Examination (MMSE), and Montreal Cognitive Assessment (MoCA), may not be sufficiently sensitive or specific to definitively diagnose cognitive impairment [34]. Mild cognitive impairment also identifies those who are most likely to benefit from heart function clinics as well. Therefore, there is a need for additional approaches to evaluate cognition. We found that the Creyos cognitive function testing was feasible based on the observed participant completion rate, the time it took for the participants to complete the tests, and the participant experience. We also explored how our data compared to normative data. Interestingly, our participants with HF did worse on some tests and better on others when compared to age- and sex-matched Creyos normative scores. Across the cognitive domains, the most pronounced impairments for the participants with HF were reasoning ability and verbal processing skills. However, when compared to the normative data, the participants with HF did better in the STM domain. Caution is warranted in the interpretation of these specific cognitive findings because the HF cohort was a convenience sample, and the Creyos normative scores were selected in a different manner. Regardless, our feasibility study does suggest that the Creyos platform may be a valuable tool in future research on cognition in patients with HF.

The results of this pilot study aligned with other studies exploring cognitive function among patients with HF. For example, in one systematic review, authors highlighted that the participants with HF did worse than those without HF on global cognition tests [17]. Studies that also focused on specific domains, such as language, also showed that those with HF had worse results than those without HF [17,18]. In our study population, the type of HF by EF had no significant impact on cognitive function, which is consistent with other studies on patients with HF [18,19]. Patients with HFrEF performed better in Digit Span, a test measuring verbal ability. However, we did not adjust for age, which may explain this finding. Other feasibility studies exploring the use of cognitive assessment tests among the population with HF, using other instruments, also concluded that an administration time of approximately 40 minutes was appropriate [20]. Among studies of cognitive function in a stable population with HF, this study is the only one to use a digital tool, instead of paper and pen, providing the opportunity to explore the feasibility of new online methods. As compared with other studies that have also explored and established the feasibility of using Creyos in other patient groups, our study had a high recruitment rate and completion rate [16,21]. This may have been because the participants were only expected to complete the tests once, unlike a similar feasibility study [21], nor were they under the stress of preparing for cardiac surgery or navigating busy clinical care schedules as with other studies [16,21].

Easy access to an iPad for those who did not have the necessary technology may also have improved accessibility and our study completion rates compared to previous studies [21]. Having the option to complete the study at home may have increased the feasibility of the tests, as in a similar study, the participants were responsive to that option [35]. However, having the option to do it at home without a member of the research team, there may be some distractions and assistance from others, impacting participant performance [35].

Compared to a study that explored the feasibility of Creyos among patients with various illnesses, our participants did well when tested for STM and even better than the normative population [16]. Our results for STM also differed from another study that focused on patients with HF [22]. However, one study highlighted that stable participants with HF did have STM that was like healthy controls, unlike decompensated participants with HF, although this may highlight selection bias [23]. Impairments in reasoning and verbal ability may impact HF management and outcomes. The patients may have challenges with managing medications and appointments, and there may also be communication barriers [10,11]. Research and newly developed guidelines highlight the need for screening and options for interventions [10,11]. By obtaining a high recruitment and completion rate and providing options for the participants to complete the study (i.e., in person or remotely) and having a single assessment time point, our results built upon previous research and strengthen support for the use of this tool in future studies among patients with HF.

This was the first study that used Creyos within a population with HF. There were some statistically significant results, highlighting the potential connections between HF and cognitive impairment. This study also revealed ways to improve the application of this tool in future work. For example, the participants were given the option to complete the study in person and receive help from a member of the research team by accessing the tests or clarifying instructions. In future studies, the participants could be encouraged to complete the study in person to allow for timely completion and reduce the number of reminders from the research team. Further, individuals without technology and/or technological savviness can more easily participate as the instruments and support are available. This work also highlighted the importance of having a PWLE as part of the research team, as their insights into various project aspects were invaluable. Further, the participants completed a comprehensive evaluation survey. Existing studies rarely collect such feedback from the participants about their experience with this tool [16,21]. With the implementation of feedback from the participants, the findings support the use of the digital Creyos cognitive assessment tool for future research among stable patients with HF. There are implications of technological literacy barriers for scaling digital assessments across the broader population with HF. Barriers include older age, difficulty with using technology, and financial concerns, potentially impacting vulnerable populations [36]. However, technology and emerging AI techniques, such as ones that analyze speech and language function, may reduce the need for high levels of technological literacy [10].

One of the main study limitations was the small sample size, which impacted the generalizability of the results and statistical power. By having small sample sizes, we could not detect the true differences between the HFrEF and HFpEF/HFmEF. We also used convenience sampling; thus, our sample of patients with HF may not be representative of the entire population with HF. However, our study does demonstrate that the Creyos battery can be used to obtain meaningful measurements of cognitive function and represents a viable option for evaluating cognition in this at-risk patient group. Further, only seven (23%) participants with HF were women, which limited a robust comparison by sex. There is still a need to further explore the relationship between sex and cognitive function in patients with HF. One study showed that men performed worse than women on cognitive tests [37]. Sex differences in the heart-brain connection may be due to sex hormones, systemic inflammation, and neurohumoral activity [38]. Additionally, conclusions about feasibility may not apply equally to at-home and in-person administration. We could not fully analyze at-home and in-person administration. There were also individuals who withdrew after consenting or after completing a few of the tests due to various barriers such as time and technological literacy, indicating another study limitation.

To improve the quality of the data, future studies should also collect information about participant education levels, which is another factor that may influence cognitive function throughout an individual’s lifetime [24]. In addition, our ability to age-match with the Creyos normative scores was limited, as only age ranges (e.g., 60-69) were provided, impacting our ability to compare our patient data to a generally healthy population. Since we were unable to include concentration in our analysis, our findings may not capture the full scope of potential cognitive effects. Another limitation is that this study did not follow the participants longitudinally. We also acknowledge potential limitations of using normative data instead of a dedicated control group and the potential bias introduced by cardiology patients without HF. Addressing some of these limitations in future studies would help to improve the feasibility of the study and the quality of the data. In the future, the role of the PWLE can also be further systematized by formalizing role descriptions, developing an ongoing engagement plan, offering training and support, and evaluating the experience of PWLEs on the team. Additionally, using resources such as the Pride in Patient Engagement in Research (PiPER) toolkit may help guide PWLE participation [39].

## Conclusions

As more work is being done to explore the connection between cognition and HF, it is important to consider the feasibility of online cognitive tests for this population. Overall, our data showed that Creyos is a feasible cognitive assessment tool to use in patients with HF, who are of an older demographic, as it can be administered when the participants have the required skills and resources. Our study shows real-world application (at home and in person at a heart function clinic). It also highlights the importance of testing the feasibility of a digital tool before moving on to a larger observational study or randomized controlled trial (RCT). Our results demonstrate that the Creyos test scores have the refinement necessary to be used in clinical trials as a cognitive outcome or baseline covariate. However, longitudinal follow-up or repeated assessments are needed to establish the reliability and clinical utility of Creyos in HF. Most participants found that the tests were easy to access and complete, and the time required for completion of all 12 tests was generally not seen as a burden. The results also showed valuable insights into the cognitive function of individuals living with HF. Future studies should apply this tool with the suggested improvements this study highlighted, in a larger and more diverse sample size, to further explore the relationship between HF and cognitive function.

## Appendices

 Table 8 shows the participant demographic form.

**Table 8:** Participant demographic form.

Question	Response
What year were you born?	
What is your gender? Check one only.	Female
	Intersex
	Male
	Trans: female to male
	Trans: male to female
	Prefer not to answer
	Do not know
How would you rate your level of English fluency? Check one only.	Fluent
	Limited fluency
	I do not speak English
	Prefer not to answer
Which of the following best describes your racial or ethnic group? Check one only.	Asian: East (e.g., Chinese, Japanese, and Korean)
	Asian: South (e.g., Indian, Pakistani, and Sri Lankan)
	Asian: South East (e.g., Malaysian, Filipino, and Vietnamese)
	Black: African (e.g., Ghanaian, Kenyan, and Somali)
	Black: Caribbean (e.g., Barbadian and Jamaican)
	Black: North American (e.g., Canadian and American)
	First Nations
	Indian: Caribbean (e.g., Guyanese with origins in India)
	Indigenous/Aboriginal: not included elsewhere
	Inuit
	Latin American (e.g., Argentinean, Chilean, and Salvadoran)
	Métis
	Middle Eastern (e.g., Egyptian, Iranian, and Lebanese)
	White: European (e.g., English, Italian, Portuguese, and Russian)
	White: North American (e.g., Canadian and American)
	Mixed heritage (e.g., Black, African; White, North American) (please specify): _____________
	Other(s) (please specify): _____________
	Prefer not to answer
	Do not know

 Table 9 shows the participant evaluation survey.

**Table 9:** Participant evaluation survey.

Item	Response Options
It was easy to access the test and get started.	Strongly disagree
	Disagree
	Neither disagree nor agree
	Agree
	Strongly agree
I found this assessment tool complicated.	Strongly disagree
	Disagree
	Neither disagree nor agree
	Agree
	Strongly agree
I thought this tool was easy to use.	Strongly disagree
	Disagree
	Neither disagree nor agree
	Agree
	Strongly agree
I needed the support of a more technical person to be able to do this test.	Strongly disagree
	Disagree
	Neither disagree nor agree
	Agree
	Strongly agree
I think most people would learn to use this tool very quickly.	Strongly disagree
	Disagree
	Neither disagree nor agree
	Agree
	Strongly agree
I found the tool very awkward to use.	Strongly disagree
	Disagree
	Neither disagree nor agree
	Agree
	Strongly agree
I felt very confident using the tool.	Strongly disagree
	Disagree
	Neither disagree nor agree
	Agree
	Strongly agree
I was able to complete all the tests within a reasonable amount of time.	Strongly disagree
	Disagree
	Neither disagree nor agree
	Agree
	Strongly agree
I was able to follow the instructions easily to get started on each test activity.	Strongly disagree
	Disagree
	Neither disagree nor agree
	Agree
	Strongly agree
I needed to learn a lot of things before I could get going with each test activity.	Strongly disagree
	Disagree
	Neither disagree nor agree
	Agree
	Strongly agree
Is there anything the research team could do to make it easier for you to take the Creyos tests?	
Do you have any other comments about the online Creyos tests?	
